# A Comparative Analysis on the Structure and Function of the *Panax notoginseng* Rhizosphere Microbiome

**DOI:** 10.3389/fmicb.2021.673512

**Published:** 2021-06-09

**Authors:** Ling Kui, Baozheng Chen, Jian Chen, Rouhallah Sharifi, Yang Dong, Zhanjiang Zhang, Jianhua Miao

**Affiliations:** ^1^School of Pharmacy, Jiangsu University, Zhenjiang, China; ^2^College of Food Science and Technology, Yunnan Agricultural University, Kunming, China; ^3^International Genome Center, Jiangsu University, Zhenjiang, China; ^4^Department of Plant Protection, College of Agriculture, Razi University, Kermanshah, Iran; ^5^College of Biological Big Data, Yunnan Agricultural University, Kunming, China; ^6^Guangxi Key Laboratory of Medicinal Resources Protection and Genetic Improvement, Guangxi Botanical Garden of Medicinal Plants, Nanning, China; ^7^State Key Laboratory for Conservation and Utilization of Bio-Resources in Yunnan, Yunnan Agricultural University, Kunming, China; ^8^School of Pharmacy, Guangxi Medical University, Nanning, China

**Keywords:** *Panax notoginseng*, rhizosphere microbiome, microbial diversity, metagenome, planting patterns

## Abstract

*Panax notoginseng*, an important Chinese medicinal herb, can be mainly cultivated in two planting patterns, cropland planting (DT) and understory planting (LX). We speculate that the rhizosphere microbiome may vary in DT and LX and may play an important role in promoting the growth and health of *P. notoginseng*. In the present study, culture-independent Illumina HiSeq was employed to investigate the rhizosphere bacteria and fungi under DT and LX planting patterns. Predominant phyla include *Proteobacteria*, *Acidobacteria*, *Actinobacteria*, *Gemmatimonadetes*, and *Ascomycota* in the two planting patterns. DT has higher alpha diversity index than LX. The predominant LX-core genera include *Bradyrhizobium, Streptomyces*, *and Actinomadura*, and the predominant DT-core genera include *Sphingomonas*, *Variovorax*, and *Novosphingobium*. Total relative abundance of the disease-suppression phylum (*Proteobacteria*, *Firmicutes*, and *Actinobacteria*) and the potential plant growth-promoting rhizobacteria (PGPR) were both significantly higher in LX than in DT. We also identified over-presented microbial functional traits mediating plant–microbe and microbe–microbe interactions, nutrition acquisition, and plant growth promotion in *P. notoginseng* rhizosphere. Our findings provide a valuable reference for studying beneficial microbes and pathogens of *P. notoginseng* planted in DT and LX.

## Introduction

*Panax notoginseng (P. notoginseng)* is an important Chinese medicinal plant commonly known as Sanqi or Tianqi. It belongs to the family *Araliaceae* (Guo et al., 2010). There is a very high global demand for this species due to its antihypertensive, antithrombotic, anti-atherosclerotic, anti-tumor, anti-oxidant, and hepatoprotective activities (Zhou et al., 2009; Yang et al., 2015; Dong et al., 2019; Li et al., 2019; Liu et al., 2019; Zhang et al., 2019a; Tang et al., 2020). Recently, more than 4 million hectares of land are cultivated annually in China for this species (Shoyama et al., 1997; Deborah et al., 2005; Wei et al., 2015). After 1–3 years of cropping, root rot is prevalent in this species (Sun et al., 2015; Yang et al., 2015; Zhang et al., 2019b).

Currently, it is grown only in southern China, typically around Wenshan City of Yunnan Province and Baise Ccity of Guangxi Province (Guo et al., 2010). Besides the root rot and limited suitable cropland, the loss of genetic diversity, and degeneration of germplasm associated with *P. notoginseng* also hamper its sustainable supply in the global market (Zhou et al., 2005).

Studies have revealed several factors, which limit its cropping, like the alteration of its soil microbiota (Dong et al., 2016; Fan et al., 2016; Miao et al., 2016; Tan et al., 2017a), autotoxicity caused by allelochemicals (Sun et al., 2008; Yang et al., 2015), and enhanced soil salinization and acidification as well as loss of nutrients from the soil (Liu et al., 2013). To mitigate these problems, biological control, chemical control, and crop rotation have been found effective (Klose et al., 2006; Song et al., 2014; Wang et al., 2016).

Cultivation of Chinese medicinal herbs as understory plants provides a healthy microenvironment for their optimum growth (Yan et al., 2016). It also limits the costs associated with labor, fertilizer application, and shading (Zhou et al., 2016). The microbiome of the rhizosphere influences plant growth (Kwak et al., 2018; Xu J. et al., 2018). The structure and composition of the rhizosphere microbiome are, in turn, determined by host species, location, planting pattern, and growth period (Peiffer et al., 2013; Edwards et al., 2015; Jin et al., 2017; Fan et al., 2019). The advent of high-throughput sequencing (HTS) technology provides an effective platform to decipher the structures and functions of the rhizosphere microbiome (Xu J. et al., 2018; Xiao et al., 2019). The whole-genome shotgun sequencing has been applied to study the rhizobiome of multiple crops such as rice, millet, corn, wheat, sugarcane, Populus, and grapevine (Daniel, 2005; Knief et al., 2012; Edwards et al., 2015; Fan et al., 2016; Knight et al., 2018; Kwak et al., 2018; Xu J. et al., 2018).

The studies on the rhizosphere microbiome of *P. notoginseng* are scarce, and a comparative evaluation of the rhizobiome composition in main cropland and understory cultivation is lacking. This study was therefore carried out to decipher the microbiome variations between the understory plantations and main croplands of *P. notoginseng* using a high-throughput metagenomic approach in the Wenshan City of Yunnan Province and the Baise City of Guangxi Province. This study holds the promise to link the better efficiency of *P. notoginseng* in the understory plantations with its specific rhizosphere microbiome when compared with the main cropland. We used a metagenomic approach to reveal the rhizosphere microbiome of *P. notoginseng* in the Wenshan City of Yunnan Province and the Baise City of Guangxi Province.

## Materials and Methods

### Sample Collection

**FIGURE 1:**
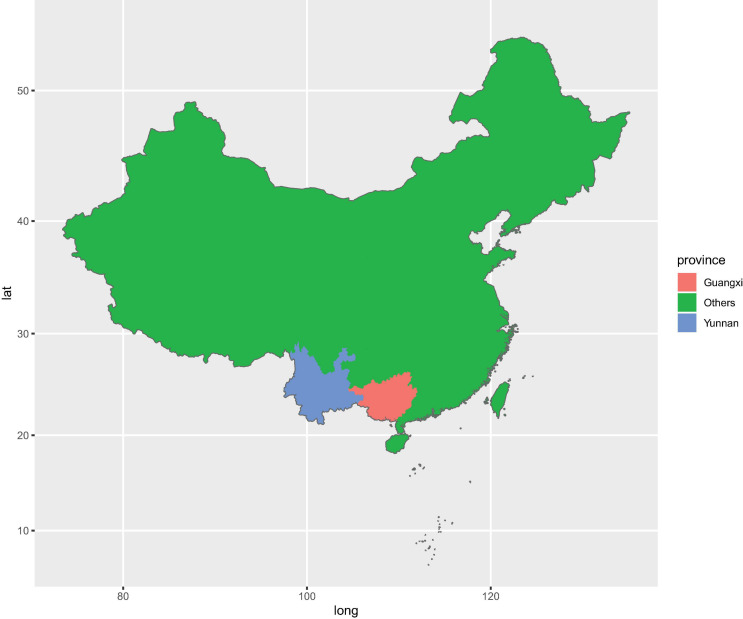
Geographic distribution of sampling sites across the main producing areas of *Panax. notoginseng.*

For sampling, we chose four farms where *P. notoginseng* was grown for the first time since 2015. These four farms were located in Wenshan City (WS) (Yunnan Province, China) and Baise City (BS) (Guangxi Province, China) (Figure 1 and Supplementary Figure 1). In order to ensure the consistency of sampling time, samples were collected from four farms through four groups at the same day. Meanwhile, sampling 1–5-year-old *P. notoginseng* also weakens the influence on microbial community caused by the growth period. We used the protocol developed by Bulgarelli et al. (2015) to collect the rhizosphere soil and the corresponding bulk soil samples. From each plant, three to five fine roots (approximately 0.5–1-mm diameter, 3–5-cm length) from a depth of 5–15 cm were collected. The soil closely attached to the roots was washed using PBS buffer. The washed soil from the same plant was then pooled in a 50-ml Falcon tube and constitutes the rhizosphere soil. Similarly, corresponding bulk soil was collected, approximately 10–20 cm underground and 0.5 m away from the *P. notoginseng* clusters, from each farm and pooled together into a 50-ml Falcon tube as one sample. The rhizosphere soil and the corresponding bulk soil were stored at 4°C until subsequent DNA extraction.

In total, 122 rhizosphere soil samples and 13 bulk soil samples were harvested from Wenshan City (WS) and Baise City (BS). Around 57 samples were harvested from WS, with 26 understory (LX) rhizosphere soil samples (WSLX) and three understory bulk soil samples (WSLXCK), 25 cropland (DT) rhizosphere soil samples (WSDT), and three cropland bulk soil samples (WSDTCK). Similarly, 78 samples were harvested from BS, with 44 understory (LX) rhizosphere soil samples (BSLX), three understory bulk soil samples, 27 cropland (DT) rhizosphere soil samples (BSDT), and four cropland bulk soil samples (BSDTCK). The details of the sampling sites are presented in Supplementary Table 1.

### DNA Extraction and Sequencing

We used EZNA soil DNA extraction kit (OMEGA Bio-Tek, Inc., Norcross, GA, United States) for extracting DNA from the collected samples. The quality of the extracted DNA was assessed through an agarose gel (1%) electrophoresis and NanoPhotometer^®^ spectrophotometer (IMPLEN, CA, United States). The concentration was measured through Qubit^®^ DNA Assay kit using Qubit^®^ 2.0 Fluorometer (Life Technologies, CA, United States). A total of 700 ng of DNA was used for library preparation. Sequencing libraries were generated using NEB ^®^ Ultra DNA Library Prep kit for Illumina^®^ (NEB, United States), following the manufacturer’s recommendations. The index codes were added to attribute sequences to each sample.

Briefly, DNA was purified using AMPure XP system (Beckman Coulter, Beverly, United States). Subsequent to the adenylation of 3′ ends of the DNA fragments, the NEBNext Adaptor with a hairpin loop structure was ligated. Next, electrophoresis was used to select DNA fragments of 350–400-bp size range. Then 3 μl of USER enzyme (NEB, United States) was used with size-selected and adaptor-ligated DNA fragments at 37°C for 15 min, followed by 5 min at 95°C before PCR. PCR was performed with Phusion High-Fidelity DNA Polymerase, universal PCR primers, and index (X) primer. Finally, PCR products were purified (AMPure XP system), and library quality was assessed on the Agilent Bioanalyzer 2100 system. The clustering of the index-coded samples was performed on a cBot Cluster Generation System using HiSeq 4000 PE Cluster kit (Illumia), as per the manufacturer’s instructions. Totally, 135 DNA libraries were sequenced on an Illumina Hiseq 4000 platform and 1,222.690 Gb of raw data was obtained, including 51 rhizosphere soil and six bulk soil samples in WS and 71 rhizosphere soil and seven bulk soil samples in BS.

### Data Analysis

The DNA from the rhizosphere and their corresponding bulk soil samples of *P. notoginseng* were sequenced at Novogene—Tianjing, China (Quince et al., 2017). We used SOAPnuke v2.0 to remove the adaptor sequences and low-quality reads harboring >10% N bases or < 40% bases with scores exceeding 38 (Chen et al., 2018). These cleaned reads were then aligned to the *P. notoginseng* reference genome released by Zhang et al. (2017) using Bowtie2 v2.3.1 (Langmead and Salzberg, 2012). The mapped reads were classified according to the farm, bulk soil, and rhizosphere soil (Supplementary Table 2).

The assemblies were generated using Megahit v1.1.3 with the default parameters (Li et al., 2016). Subsequently, the MetaGeneMark_v1_mod implemented in Prodigal v2.6.3 was used to predict the genes on the contigs (Zhu et al., 2010). All the genes of length ≥ 100 bp were retained for downstream analysis. With the assistance of the Mmseq2 software and the Linclust algorithm, all the predicted genes were combined together and then clustered into a non-redundant geneset, using the similarity threshold at 95% (Steinegger and Soding, 2017). For the taxonomic and functional information of the unigenes, their protein sequences were aligned against the NCBI non-redundant database and Kegg Orthology (KO) database, using DIAMOND at an *E*-value threshold of 1e^–5^ (Buchfink et al., 2015). Moreover, the cleaned reads were aligned against the geneset to obtain the abundance profiles (read count matrix) usingBowtie2 v2.3.1 (Langmead and Salzberg, 2012).

### Comparative Analysis Across Different Planting Patterns

We used a DESeq2 v1.30.1-based negative binomial generalized linear model to statistically assess the differences in the abundance profiles of genera, phyla, and KOs across the understory (LX) and main cropland (DT) planting patterns. First, the read count matrix was normalized through the DESeqVS method (Love et al., 2014; Tang et al., 2015), followed by a comparative analysis to detect LX-enriched genera, DT-enriched genera, and enriched KOs using DESeq2. We used the criteria that if taxa or a KO is present in more than 75% of the samples, in each of the different planting patterns, they were considered as core taxa or core KOs (Xu J. et al., 2018). For visually assessing the relative abundance of the enriched taxa and the functional traits in the rhizosphere, we used the Pheatmap R package. To clearly demonstrate the differences among the core taxa and their functional traits in different planting patterns, the relative abundance was calculated based on the number of mapped reads.

### Weighted Gene Co-expression Network Analysis

Weighted gene co-expression network analysis (WGCNA) was performed in prokaryotes and fungi, respectively. Classified prokaryote samples were divided into eight groups according to planting location and pattern, including WSLX, WSDT, BSLX, BSDT, WSLXCK, WSDTCK, BSLXCK, and BSDTCK. Fungi samples also follow this classification criteria. Construction of weighted co-expressed networks and identification of co-expression modules were carried out using the “WGCNA” R package with default parameters, the input file is the count table (Supplementary Table 3). In order to ensure a scale-free network, the power of β = 11 (scale-free R2 = 0.8) was selected as the soft thresholding, and MEDissThres was set as 0.25 to merge similar modules. After that, nodes were screened by the criteria MM > 0.87 for pink module in prokaryotes and MM > 0.02 for turquoise module in fungi. Then the nodes were inputted into Cytoscape to visualize the co-expression network, and the nodes and hub genera were identified (Su et al., 2014).

## Results

### Taxonomic Profile of *Panax notoginseng* Rhizosphere Microbiome

The shotgun metagenome sequencing generated approximately 30 million 150-bp paired-end reads for each sample, accounting for more than 607.7 Gbp raw sequences. After the removal of low-quality sequences (Supplementary Table 2), the *de novo* assembly was performed through Megahit 1.1.3. A total of 149,436,529 contigs were obtained in the final assembly, constituting a total length of 103.6 Gb. Approximately 193 million metagenes were predicted from the contigs using MetaGeneMark_v1_mod. These metagenes were then clustered into approximately 164 million non-redundant genes (unigenes). In total, 164,264,054 unigenes were generated in our study and used for downstream analysis.

**FIGURE 2:**
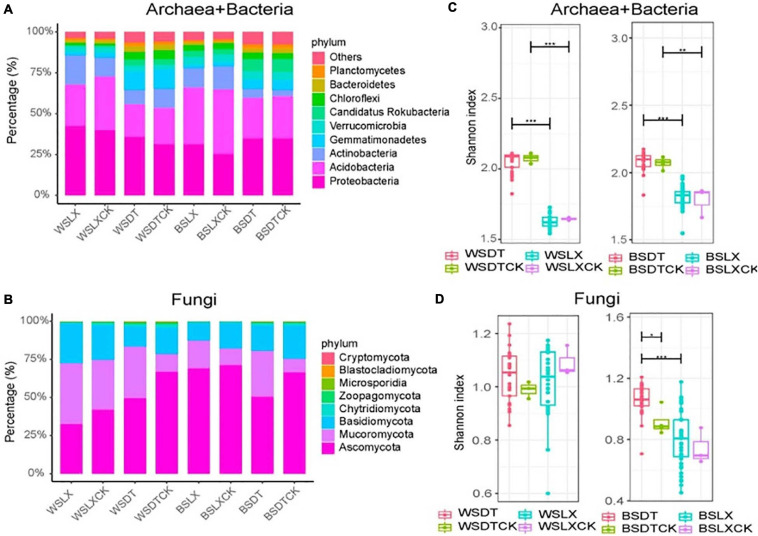
Taxonomic distribution and diversity comparisons in *P. notoginseng* rhizosphere (*n* = 122) and bulk soil (*n* = 13) microbiomes across different sites. **(A,B)** Phyla-level distributions of prokaryotes and fungi in the bulk soil and rhizosphere samples based on metagenomic data. **(C,D)** Alpha diversity comparison between the bulk soil and rhizosphere samples from each location based on the Shannon index using the metagenomic data; *P < 0.05; **P < 0.01; ***P < 0.001. One-sided *t*-test; center value represents the median of the Shannon index. WSDT, rhizosphere soil of cropland planting in Wenshan city; WSDTCK, bulk soil of cropland planting in Wenshan City; WSLX, rhizosphere soil of understory planting in Wenshan City; WSLXCK, bulk soil of understory planting in Wenshan City; BSDT, rhizosphere soil of cropland planting in Baise City; BSDTCK, bulk soil of cropland planting in Baise City; BSLX, rhizosphere soil of understory planting in Baise City; BSLXCK, bulk soil of understory planting in Baise City.

Taxonomic annotations were assigned to 62.4% of the unigenes, among which 99.55% were classified into 165 prokaryotic phyla (including bacteria and archaea) and 0.43% into 31 eukaryotic phyla (Supplementary Figure 2). A negligible fraction (0.014%) of the annotated unigenes were classified as viral, indicating potential annotation biases that resulted in the underestimation of eukaryotic and viral communities (Leach et al., 2017). We focused our study on the comparative analysis of the prokaryotic and fungal rhizosphere microbiomes in the two planting patterns (DT and LX). The dominant prokaryotic (bacterial and archaeal) phyla found in the *P. notoginseng* rhizosphere and bulk soil included *Proteobacteria* (34.5%), *Acidobacteria* (28.6%), *Actinobacteria* (10.5%), *Gemmatimonadetes* (5.3%), and *Verrucomicrobia* (4.2%). These together constituted about 83.1% of the prokaryotic unigenes (Figure 2A and Supplementary Table 3-1). Fungal unigenes were primarily associated with the phyla *Ascomycota* (57.8%), *Mucoromycota* (22.1%), and *Basidiomycota* (17.8%), which together accounted for 97.7% of the fungal unigenes (Figure 2B and Supplementary Table 3-2).

### Estimation of the Microbial Diversity

The alpha diversity (α-diversity) analysis was estimated based on the Shannon index. We observed a significant difference in α-diversity between different planting patterns (DT and LX) in *P. notoginseng* rhizosphere. DT showed a higher α-diversity than LX, except for the fungal taxa in WS. A similar trend was shown by α-diversity of bulk soil microbial community, but little significant difference in α-diversity was observed between the rhizosphere and bulk soil samples, except for the fungal taxa at BSDT (Figures 2C,D).

**FIGURE 3:**
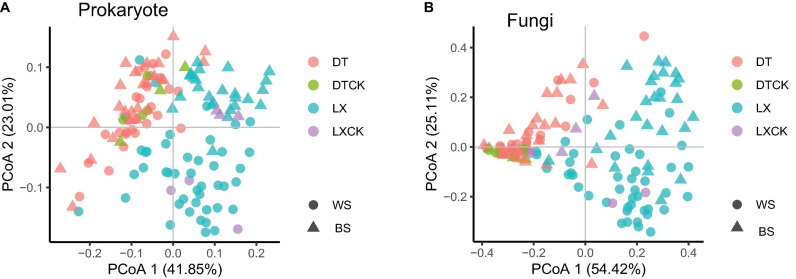
**(A,B)** Principal coordinate analysis (PCoA) of prokaryote and fungi based on the Bray–Curtis distance between the bulk soil (*n* = 13) and rhizosphere soil (*n* = 122) for each location using the metagenomic data. WS, Wenshan City; BS, Baise City; DT, cropland planting rhizosphere soil; DTCK, cropland planting bulk soil; LX, understory planting rhizosphere soil; LXCK, understory planting bulk soil.

Furthermore, the principal coordinate analysis (PCoA) based on the Bray–Curtis distance (β-diversity) revealed that the planting pattern was the principal factor in shaping the composition of the microbial communities (Figures 3A,B). Moreover, no significant variation among the microbial taxa between the rhizosphere and the bulk soil samples was revealed by the β-diversity analysis (Figures 3A,B). These results were corroborated by the permutation multivariate analysis of variance (PERMANOVA), which was according to the Bray–Curtis distance metric. PERMANOVA showed a significant variation in prokaryotic taxa (25.7%, *P* = 0.001) and fungal taxa (34.5%, *P* = 0.001) between LX and DT (Supplementary Table 4). The planting location appeared as the second-largest source to shape the microbial community difference (Supplementary Table 4).

The enrichment analysis of the microbial phyla, based on their relative abundance, revealed that 58 prokaryotic phyla and seven fungal phyla were enriched in the LX rhizosphere microbiome, whereas 73 prokaryotic phyla and one fungal phylum were enriched in the DT rhizosphere microbiome (corrected *P*-value < 0.05, DESeq2, Supplementary Table 5-1). Moreover, no significant variation in the relative abundance of prokaryotic or fungal phyla was observed between the rhizosphere and bulk soil microbial phyla (corrected *P*-value ≥ 0.05, DESeq2, Supplementary Table 5-2). Similarly, the enrichment analysis of the microbial genera revealed 902 prokaryotic genera and 251 fungal genera as enriched in the LX rhizosphere microbiome, whereas 1,087 prokaryotic genera and 27 fungal genera were enriched in the DT rhizosphere microbiome (corrected *P*-value < 0.05, DESeq2, Supplementary Table 6-1). These enriched genera belonged to the main prokaryotic phyla like *Proteobacteria*, *Acidobacteria*, *Actinobacteria*, and *Verrucomicrobia* and the main fungal phyla like *Ascomycota* and *Mucoromycota* (Figures 2A,B and Supplementary Tables 5, 6). In comparison with the bulk soil microbiome, the rhizosphere microbiome contained only two enriched prokaryotic genera, *Nitrosopumilus* and *Wenzhouxiangella* along with depletion of three prokaryotic genera and two fungal genera (corrected *P*-value < 0.05, DESeq2, Supplementary Table 6-2).

### Core Genera Evaluation

**FIGURE 4:**
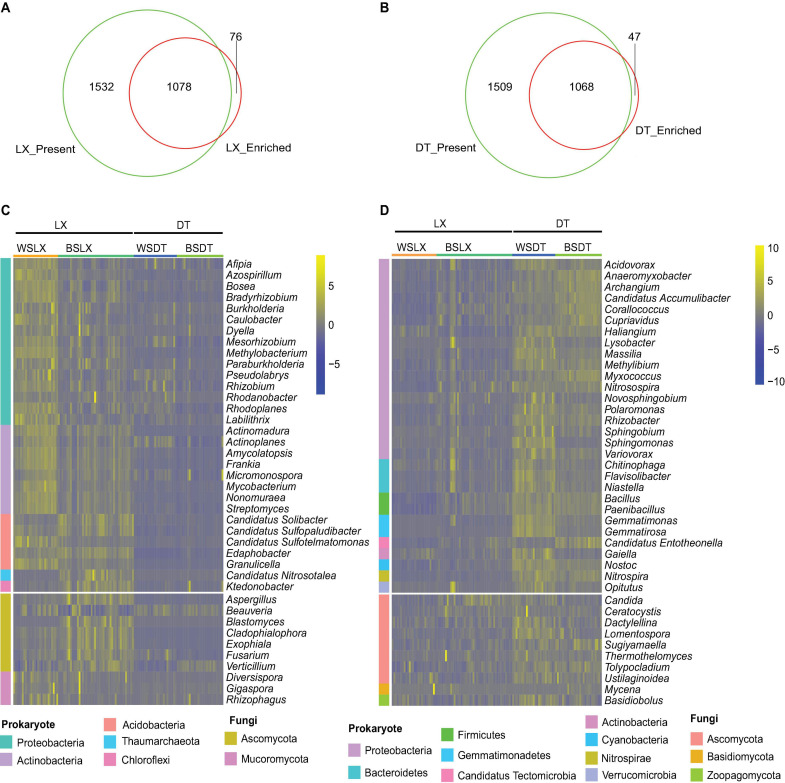
Characterization of the core *P. notoginseng* rhizosphere microbes in cropland planting (*n* = 52) and understory planting (*n* = 70) patterns. **(A)** Venn plot depicting the number of core rhizosphere genera based on the presence rate (>75%) in all understory planting samples and the genus have a higher relative abundance in understory planting than in cropland planting. **(B)** Venn plot depicting the number of core rhizosphere genera based on the presence rate (>75%) in all cropland planting samples and the genus have a higher relative abundance in cropland planting than in understory planting. **(C,D)** Relative abundances of the 30 prokaryotes and 10 fungi, most relatively abundant core rhizosphere genera in cropland planting and understory planting patterns based on metagenomic data. Scale, relative abundance of genus at row normalization by removing the mean (centering) and dividing by the standard deviation (scaling). The color from blue to yellow represents a relative abundance of each taxa from low to high. WS, Wenshan City; BS, Baise City; DT, cropland planting rhizosphere soil; LX, understory planting rhizosphere soil.

Using the aforementioned criteria, the core genera (genera present in more than 75% of all samples and with enriched relative abundance) in the *P. notoginseng* rhizosphere of LX and DT planting patterns were evaluated. Around 875 prokaryotic and 203 fungal genera were found as core genera in LX-rhizosphere and 1,051 prokaryotic and 17 fungal genera as core genera in DT-rhizosphere of *P. notoginseng* (Figures 4A,B and Supplementary Table 6). The core rhizosphere genera were mainly affiliated with the prokaryotic phyla *Proteobacteria*, *Actinobacteria*, and *Acidobacteria* as well as with the fungal phyla *Ascomycota* and *Mucoromycota* (Figure 4C). *Bradyrhizobium*, *Burkholderia*, and *Paraburkholderia* have higher relative abundance (corrected *P*-value < 0.05, DESq2) (Figures 4C,D and Supplementary Figure 5), and were over-represented in *Proteobacteria*. At the same time, both LX and DT core genera contain some inhibitors and pathogenic microbiomes, which may interfere with the quality and growth of *P. notoginseng*.

### Characteristic Analysis of Core Rhizosphere Microbiome of Panax Notoginseng in the Different Planting Patterns

**FIGURE 5:**
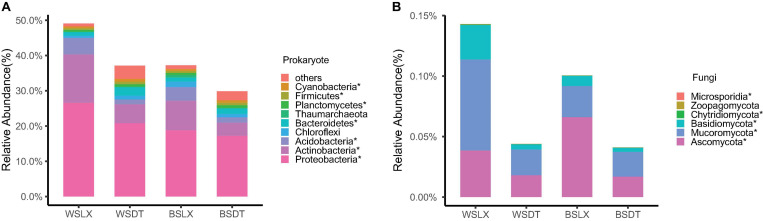
Phyla-level distributions of core rhizosphere microbiomes in cropland planting (*n* = 52) and understory planting (*n* = 70) patterns based on metagenomic data. **(A)** Detailed relative abundances of core prokaryotic rhizosphere microbiomes. **(B)** Detailed relative abundances of core fungal rhizosphere microbiomes. Asterisk indicates a significant difference with *P* < 0.05 (MW test). WSDT, rhizosphere soil of cropland planting in Wenshan City; WSLX, rhizosphere soil of understory planting in Wenshan City; BSDT, rhizosphere soil of cropland planting in Baise City; BSLX, rhizosphere soil of understory planting in Baise City.

**FIGURE 6:**
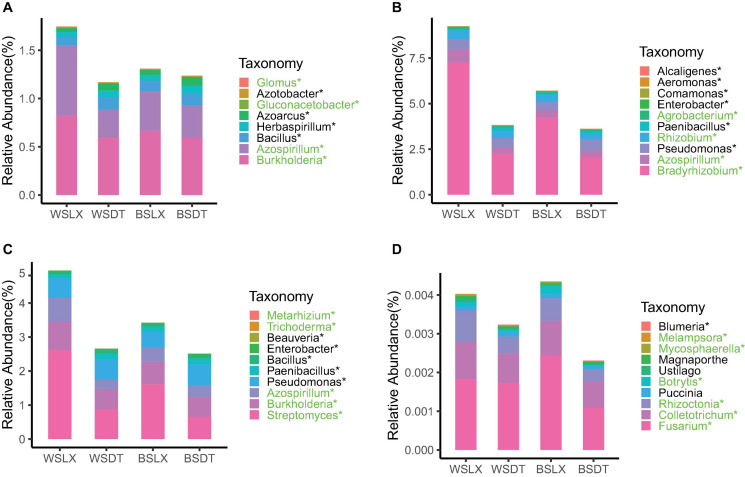
Core genera involved in plant growth-promoting rhizobacteria (PGPR) or plant pathogens in cropland planting (*n* = 52) and understory planting (*n* = 70) patterns across different sites based on metagenomic data. Detailed relative abundances of core rhizosphere microbiomes involved in **(A)** nitrogen cycle, **(B)** plant growth regulator, **(C)** biological control agent, and **(D)** plant pathogens. Asterisk indicates a significant difference with *P* < 0.05 (MW test). Green indicates the relative abundances of core rhizosphere microbiomes is significantly higher in understory planting than in cropland planting. Black indicates the relative abundances of core rhizosphere microbiomes is significantly lower in understory planting than in cropland planting. WSDT, rhizosphere soil of cropland planting in Wenshan City; WSLX, rhizosphere soil of understory planting in Wenshan City; BSDT, rhizosphere soil of cropland planting in Baise City; BSLX, rhizosphere soil of understory planting in Baise City.

Mann–Whitney U test revealed that the total relative abundance of the core rhizosphere microbiome phyla varied significantly between LX and DT planting patterns. We observed that the multiple disease-suppression prokaryotes were highly abundant in LX than DT (*P* < 0.05), among which *Proteobacteria*, *Firmicutes*, and *Actinobacteria* were important in disease suppression (Mendes et al., 2011; Figure 5A). Furthermore, 21 potential plant growth-promoting rhizobacteria (PGPR) genera were identified, among which eight genera were related to the nitrogen cycle (Nelson et al., 2016; Supplementary Table 6), 10 genera were plant growth regulators, and 10 genera were the biological control agents (Figures 6A–C). The total relative abundance of these PGPR genera was found higher in LX than DT (Figure 6D).

However, the pathogenic fungal phyla like *Basidiomycota*, *Mucoromycota*, and *Ascomycota* were also significantly higher in LX than in DT (*P* < 0.05) (Figure 5B). The potential plant pathogen genera like *Fusarium*, *Colletotrichum*, and *Rhizoctonia* were higher in LX than in DT (*P* < 0.05, MW test) (Figure 6D; Fravel et al., 2003). Besides the pathogenic nature, the genera *Fusarium* has several members, which are non-pathogenic and may act as potential biocontrol agents that could control fungal pathogens (Nel et al., 2006). Similarly, *Colletotrichum* has also been proposed as a means to promote plant growth through hormone production and phosphorus absorption under abiotic stress (Hiruma et al., 2016; Figure 6D).

### The Correlations of Microbiomes Analyzed by Weighted Gene Co-expression Network Analysis

**FIGURE 7:**
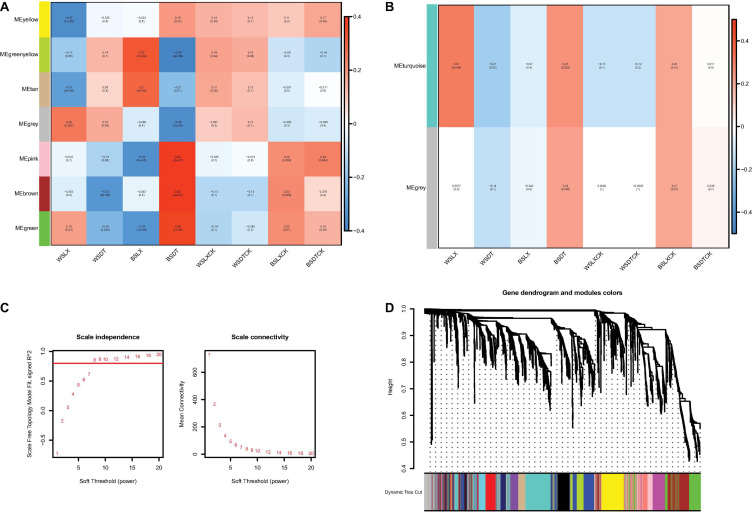
The correlations of microbiomes. Heat map of module-trait relationship in prokaryotes **(A)** and fungi **(B)**. Each column represents the co-expression module. Red color of each box represents the positive correlation between module and trait. Blue color of each box represents the negative relationships between module and trait. The darker the module color, the more significant their relationship. **(C)** Estimation of soft-thresholding values in prokaryotes. Scale independence and mean connectivity of various soft-thresholding values (β), power of β = 11 (scale free R2 = 0.8). **(D)** Dendrogram of all prokaryotes enriched according to a dissimilarity measure (1-TOM) and the cluster module colors. The first set of modules was obtained with dynamic tree cut algorithm and the correlated modules were merged together. Each branch in the figure represents one genus and every color below represents one co-expression module.

The correlations of microbiomes under different planting patterns are shown in Figure 7. For fungi, the difference of gray and turquoise module could be seen under different planting patterns or location. However, as the number of genera is small, there are fewer modules displayed in fungi (Figure 7B), and we mainly focused on prokaryotes (Figures 7A,C,D). For the prokaryotic community, seven modules were generated; the modules represented distinct differences under different planting patterns (WSLX vs. WSDT, BSLX vs. BSDT). BSDT had a significant positive correlation with most modules: green (*P* = 7E-06, *r* = 0.38), brown (*P* = 4E-07, *r* = 0.42), pink (*P* = 2E-07, *r* = 0.43), and yellow (*P* = 0.02, *r* = 0.19) modules (Figure 7A). Comparing the rhizosphere soil and bulk soil modules, a similar correlation was shown. Besides, the modules were also varied in location (WSLXCK vs. BSLXCK, WSDTCK vs. BSDTCK). The results are consistent with the trend of PERMANOVA’s analysis, which revealed that planting pattern is the main factor to shape the structure of *P. notoginseng* rhizosphere microbes, and second is the location.

**FIGURE 8:**
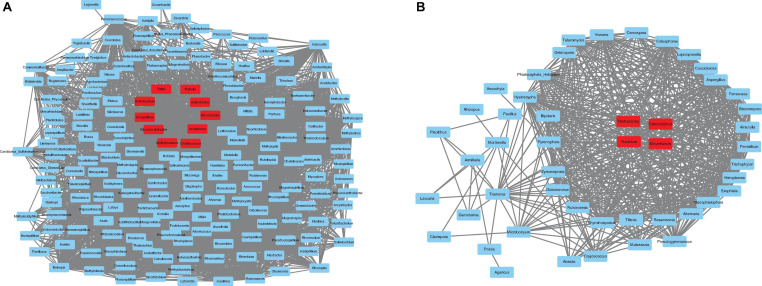
The co-expression network. **(A)** The co-expression network of the hub genera from pink module in prokaryotes, including 100 nodes. **(B)** The co-expression network of the hub genera from turquoise module in fungi, including 48 nodes. The hub genes are represented in red color.

According to the correlation results, genera from the pink module in prokaryotes and the turquoise module in fungi were extracted to generate networks (Figure 8). Nodes were screened by the criteria MM > 0.87 for the pink module and MM > 0.02 for the turquoise module. The results are shown in Figure 8. Ten hub genera were obtained in prokaryotes (Figure 8A, red color) and four hub genera in fungi (Figure 8B, red color). For prokaryotes, there are two genera, *Azospirillum* and *Gluconacetobacter*, that were highly correlated in the pink module. It is worth noting that they are also identified as PGPR’s taxa and had higher relative abundance in LX than in DT in previous analysis. Besides, two genera (*Trichoderma* and *Metarhizium*) related to biological control agent, and two genera (*Fusarium* and *Colletotrichum*) related to plant pathogen were highly correlated in the turquoise module of fungi. Similarly, these four genera had higher relative abundance in LX than in DT. It may indicate that the semi-nature planting patterns (LX) evolve a closer interaction microenvironment, which can provide us with significant reference to selectively manage microbiome in agricultural production.

### Core Functional Traits in the Different Planting Patterns of Panax Notoginseng Rhizosphere

**FIGURE 9:**
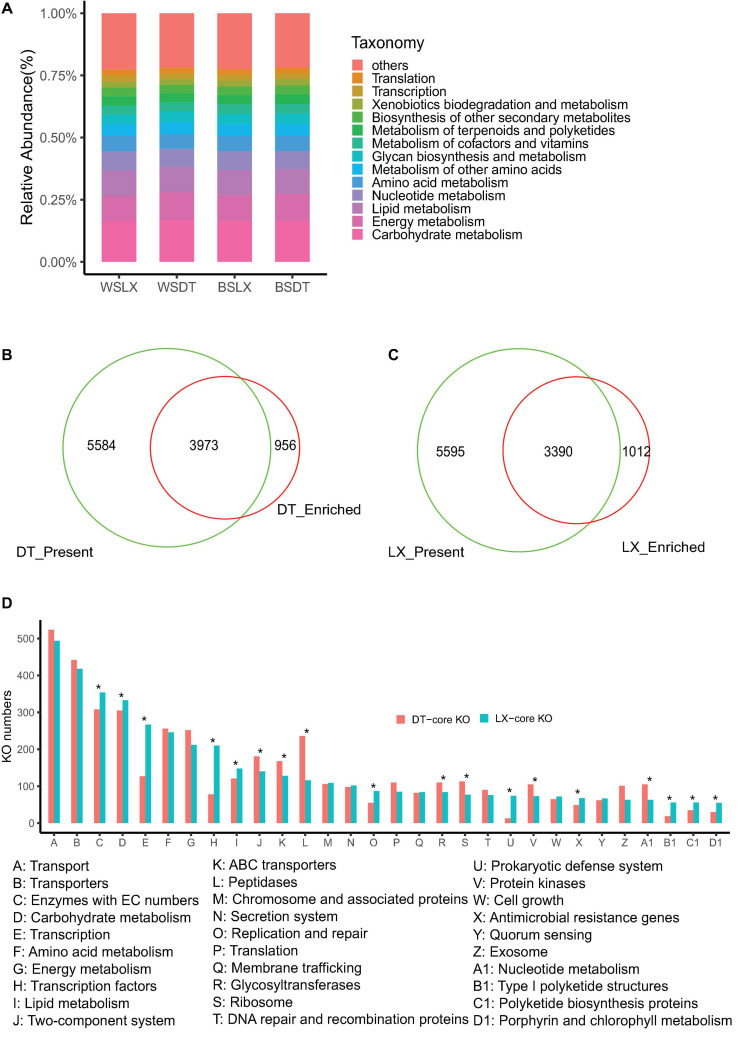
Characterization of the core function of the *P. notoginsen* rhizosphere microbiome in different planting patterns. **(A)** Functional KEGG level 2 pathway comparison of *P. notoginsen* rhizosphere microbiomes. **(B)** Venn plot depicting the number of core rhizosphere KOs based on the presence rate (>75%) in all understory planting samples, the KOs have a higher relative abundance in understory planting than in cropland planting. **(C)** Venn plot depicting the number of core rhizosphere KOs based on the presence rate (>5%) in all cropland plantin samples, the KOs have a higher relative abundance in cropland planting than in understory planting. **(D)** The distributions of the rhizosphere core KOs in the KEGG level 3 pathways. Asterisk indicates a significant difference with *P* < 0.05 (MW test). WSDT, rhizosphere soil of cropland planting in Wenshan City, WSLX, rhizosphere soil of understory planting in Wenshan City; BSDT, rhizosphere soil of cropland planting in Baise City; BSLX, rhizosphere soil of understory planting in Baise City.

The unigene sets of the *P. notoginseng* rhizosphere were 4- and 18-fold larger than that of the human gut and the global Tara oceanic microbiomes, respectively (Sunagawa et al., 2015), but it had the same order as the global citrus rhizosphere microbiome (Xu J. et al., 2018). More than 35% of (58 of 164 million) the unigenes were annotated by blast against the KEGG Orthology (KO) database, and 16,396 KOs were assigned to the annotated unigenes (Supplementary Table 7-1). These KOs were mainly associated with 41 KEGG (level 2) pathways. The carbohydrate metabolism and energy metabolism had a higher relative abundance across the different planting patterns (Figure 9A). Finally, 9,585 of 15,725 (60.9%) and 9,557 of 15,757 (60.6%) KOs were identified in at least 75% of the LX-planting and DT-planting soil microbiomes, respectively. A pairwise comparative analysis using DESq2 revealed that 5,002 and 4,929 KOs were enriched in LX-planting and DT-planting soil microbiomes, respectively. The core functional characteristics of *P. notoginseng* rhizosphere microorganisms in different planting patterns were determined, which showed that 3,990 KOs were enriched in LX and 3,973 KOs in DT (corrected *P*-value < 0.05, DESeq2) (Figures 9B–D and Supplementary Tables 7-1, 7-2).

**FIGURE 10:**
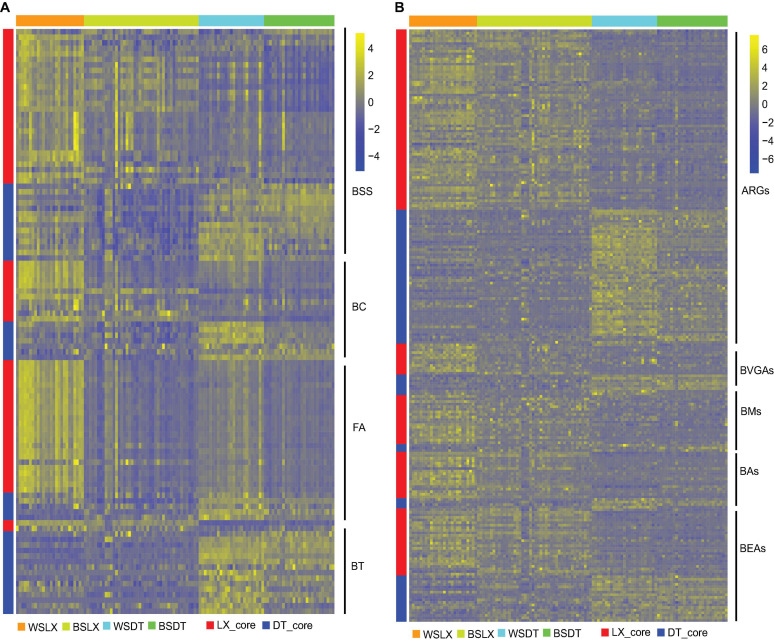
Relative abundances of core rhizosphere KOs in different planting patterns. **(A)** The relative abundance of core rhizosphere KOs involved in plant–microbe and microbe–microbe interactions. **(B)** The relative abundance of core rhizosphere KOs involved in antimicrobial resistant genes (ARGs), antibiotic synthesis genes (ASGs). Scale, relative abundance of KO at row normalization by removing the mean (centering) and dividing by the standard deviation (scaling). The color from blue to yellow represents a relative abundance of each KO from low to high. WSDT, rhizosphere soil of cropland planting in Wenshan City; WSLX, rhizosphere soil of understory planting in Wenshan City; BSDT, rhizosphere soil of cropland planting in Baise City; BSLX, rhizosphere soil of understory planting in Baise City; BSS, bacterial secretion systems; BC, bacterial chemotaxis; FA, flagellar assembly; BT, bacterial toxins; ARGs, antimicrobial resistant genes; BVGAs, biosynthesis of vancomycin group antibiotics; BMs. biosynthesis of 12-, 14-, and 16-membered macrolides; Bas, biosynthesis of ansamycins; BEAs, biosynthesis of enediyne antibiotics.

The core functional characteristics involved in plant–microbe and microbe–microbe interactions were very likely to reflect the structural traits of microbiomes harboring the *P. notoginseng* rhizosphere. MW test to compare the core KO numbers between DT and LX revealed differences in several core functions. For example, bacterial secretion systems, bacterial chemotaxis, and flagellar assembly were over-presented in LX (Figure 10A) (*P* < 0.05, MW test), whereas bacterial toxins, and two-component systems were over-presented in DT (Figure 10A and Supplementary Figure 3) (*P* < 0.05, MW test). Quorum sensing and biofilm formation had little significance (Figure 9A) (*P* ≥ 0.05, MW test). The antimicrobial resistance and antibiotic synthesis were more enriched in LX than in DT (Figure 10B). For example, KOs associated with the biosynthesis of the vancomycin group of antibiotics, biosynthesis of 12-, 14-, and 16-member macrolides, biosynthesis of ansamycins, and biosynthesis of enediyne antibiotics were significantly higher in LX than in DT (*P* < 0.05, MW test). These observations suggested that more intimate host–microbe and microbe–microbe interactions occur in the LX-planting than in the DT-planting pattern.

ABC transporters, which were mainly responsible for the transport of urea, ions, amino acids, monosaccharides, and oligosaccharides into microbial cells, were significantly more enriched in LX than in DT (Supplementary Figure 4) (*P* < 0.05, MW test). Metabolism of amino acids like glycine, serine, threonine, valine, leucine, isoleucine, lysine, and phenylalanine was significantly higher in LX than in DT (Supplementary Figure 5), whereas the KOs corresponding to alanine, aspartate, and glutamate metabolism were over-represented in DT (Supplementary Figure 6). The xenobiotics biodegradation-related KOs showed less difference between LX and DT (Supplementary Figure 6) (*P* ≥ 0.05, MW test). However, the dioxin degradation-related KOs were more enriched in DT, indicating that DT faces the problem of frequent pesticide application. The KOs corresponding to lysine, arginine, phenylalanine, tyrosine, and tryptophan biosynthesis was significantly higher in DT than in LX (*P* < 0.05, MW test) (Supplementary Figure 7), whereas the carbon fixation pathways in prokaryotes were significantly higher in LX than in DT (*P* < 0.05, MW test) (Supplementary Figure 8). A lesser number of KOs affiliated with peptidases were enriched in the LX rhizosphere microbiome (*P* ≥ 0.05, MW test) (Supplementary Figure 9). The transcription factors also showed a significant difference between DT and LX rhizosphere microbiomes. For example, the LuxR, AraC, GntR, MerR, TetR, AcrR, and LysR families of transcriptional regulators were more over-represented in LX than in DT (*P* < 0.05, MW test) (Supplementary Figure 10). Notably, such regulators were typically associated with metabolism, transport, quorum sensing, motility, stress response, and pathogenesis, which confirmed that a more intimate relationship of plant–microbe and microbe–microbe interactions appeared in LX than in DT.

## Discussion

Microorganisms dwelling in the rhizosphere of plants play an important role in their growth and development. The plants and the microbes interact with each other and influence the growth of each other. *P. notoginseng* is an important Chinese medicinal plant with high global demand, such that its demand supersedes the production. Furthermore, several factors like root rot after few years of replanting (Sun et al., 2015), less suitable cropland, depletion of genetic diversity, and germplasm degeneration limit its production (Zhou et al., 2005). It is, therefore, imperative to evaluate the factors that could enhance the survival and growth of this species. This species is grown as an understory crop besides the main cropland species. It was observed that the understory Chinese medicinal plants experience a healthy microenvironment for their optimum growth (Yan et al., 2016). The studies on the rhizobial microbiome of *P. notoginseng* are scarce, and a comparative evaluation of the rhizobiome composition in the main cropland and understory cultivation is lacking. We, therefore, were interested in evaluating the microbiome variations based on the planting pattern, i.e., main cropland (DT) and understory planting (LX), in the Wenshan City of Yunnan Province and the Baise City of Guangxi Province, using a high-throughput metagenomics approach.

We found that the predominant taxa in the host rhizosphere microbiome were mainly prokaryotes, and eukaryotes accounted for a small fraction of the sequences. This finding was corroborated by Xu J. et al. (2018). The low proportion of eukaryotic taxa in the rhizosphere of *P. notoginseng* could be attributed to the unavailability of reference genomes for most eukaryotes (Bulgarelli et al., 2015). Approximately, 60% of the unigenes were assigned to known taxa. Taxonomic annotation at the phylum level indicates that *Proteobacteria*, *Acidobacteria*, and *Actinobacteria* were dominant in the rhizosphere. Similar results were obtained by several studies (Peiffer et al., 2013; Bulgarelli et al., 2015; Edwards et al., 2015; Jin et al., 2017; Xu J. et al., 2018; Xu L. et al., 2018; Fan et al., 2019). Generally, a plant will harbor specific microbial communities in its rhizosphere from the soil reservoir to facilitate growth.

The same as the citrus, little significant difference had been revealed between *P. notoginseng* rhizosphere and bulk samples (Xu J. et al., 2018). It may be that the threshold distance rendering the microbiomes between the rhizosphere and bulk soil distinct is species dependent upon this distance, the microbiomes of rhizosphere and bulk samples are more or less homogenous in the case of *P. notoginseng*. Various studies have reported that the microbial communities existing in the soil microbial reservoir can be influenced by factors such as plant genotype, location, planting pattern, and growth period (Peiffer et al., 2013; Edwards et al., 2015; Jin et al., 2017; Fan et al., 2019). Our study revealed that the planting pattern, DT or LX, was the major factor in determining the rhizosphere microbiome profile. Both alpha and beta diversities of *P. notoginseng* rhizosphere microbiomes in DT and LX planting patterns were significantly different. Location was the second major factor. This suggests that the planting patterns shaped the unique rhizosphere microbiomes in *P. notoginseng*.

The rhizosphere microbiome profile not only influences the growth of the host plants but also may be a potential bioindicator to reflect the health status of the soil. This is established by several studies concerning the microbial communities of crops such as maize, bananas, *P. quinquefolius*, and *P. notoginseng* (Fu et al., 2017; Tan et al., 2017b; Jiang et al., 2019). Dong et al. (2016) revealed that the death rate of *P. notoginseng* and the fungal diversity had a significantly negative correlation. In our study, LX enriches more core fungal genera than DT (203 vs. 17), and the total relative abundance of the core genera that were associated with disease suppression were significantly higher in LX than in DT, such as the genera belonging to the phyla *Proteobacteria*, *Firmicutes*, and *Actinobacteria* (Mendes et al., 2011). Several genera like *Azospirillum*, *Bradyrhizobium*, *Burkholderia*, and *Streptomyces*, which are potentially beneficial (Bevivino et al., 1998; Bhattacharyya and Jha, 2012), were also significantly higher in LX than in DT. Besides, although multiple genera associated with plant pathogens were also found to be significantly higher in LX than in DT such as *Fusarium*, *Colletotrichum*, and *Blumeria*, the non-pathogenic *Fusarium* may act as a potential biocontrol agent against fungal pathogens (Nel et al., 2006). Additionally, *Colletotrichum* has been proposed to promote plant growth through hormone production and phosphorus absorption under abiotic stress (Hiruma et al., 2016). These findings seem to evidently support that LX can create a better healthy environment, but the phenomenon still needs further and more studies to confirm.

The functional traits of the rhizosphere microbiome are closely associated with the life activities of the microorganism’s host–microbe and microbe–microbe interactions (Xu J. et al., 2018). The core functional traits involved in nutrient mobilization were over-represented in DT and LX (Figure 6D), suggesting that microbiomes need access to nutrients to form the surrounding soil, i.e., plant root exudates and humus (Peiffer et al., 2013). The rhizosphere enrichment of bacterial secretion systems, chemotaxis, flagella, assembly, antimicrobial resistance, and antibiotic synthesis genes are largely depended on the coevolution of host–microbe and microbe–microbe interactions, indicating that plants will positively harbor beneficial microbes to the rhizosphere. Interestingly, we observed more core antimicrobial resistance and antibiotic synthesis genes in LX than in DT, which indicate that the long-term use of farm chemicals in DT may cause the decrease in fungi.

## Data Availability Statement

The data presented in the study are deposited in the NCBI (https://dataview.ncbi.nlm.nih.gov/objects?linked_to_id=PRJNA721004&archive=biosample) repository, accession number (PRJNA721004).

## Author Contributions

JM, ZZ, and YD designed the study. LK and BC completed the experiments, completed the data analyses, and wrote the manuscript. JC, RS, and YD edited the manuscript. All authors read and approved the final manuscript.

## Conflict of Interest

The authors declare that the research was conducted in the absence of any commercial or financial relationships that could be construed as a potential conflict of interest.
