# Supplementary material for: A Comparative Analysis on the Structure and Function of the Panax notoginseng Rhizosphere Microbiome
Source: Front Microbiol. 2021 Jun 9;12:673512. doi: 10.3389/fmicb.2021.673512 (PMC8219928; doi:10.3389/fmicb.2021.673512)
Supplement: Supplementary file 1 [file Data_Sheet_1.PDF]

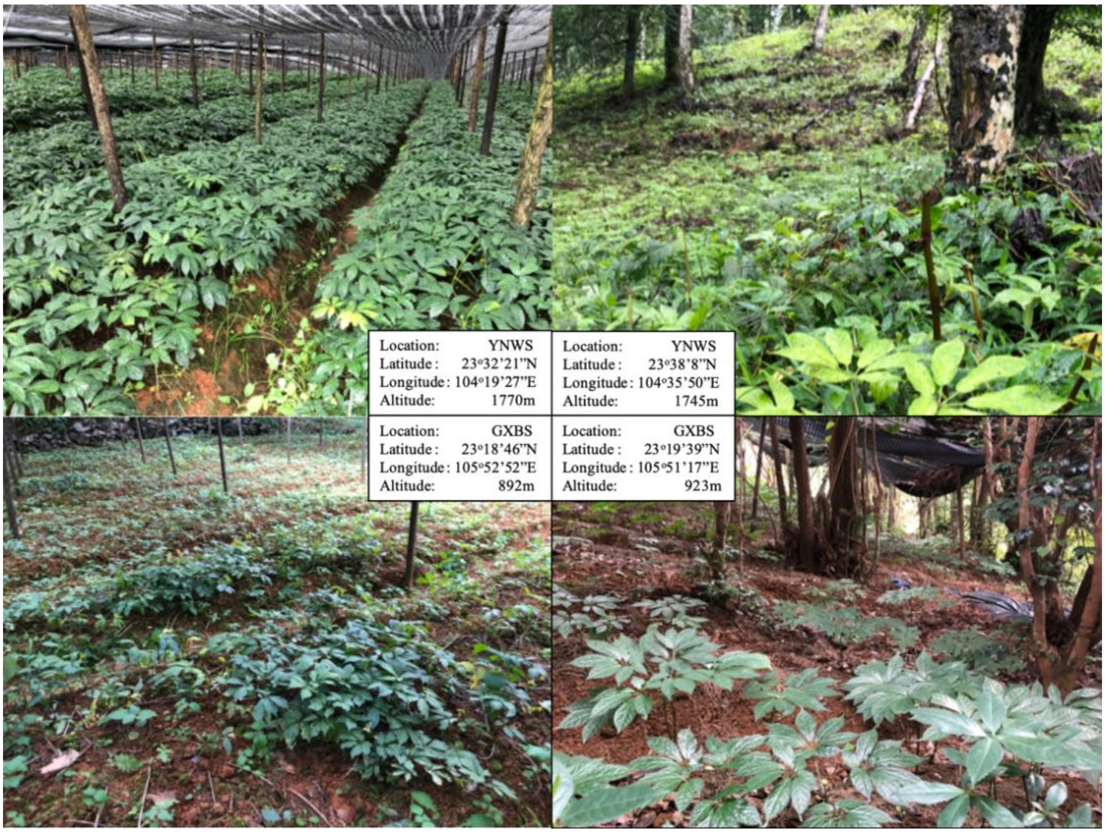

Supplementary Figure 1 The locaton information of four farms used for collecting rhizosphere microbes of *Panax. notoginseng*.

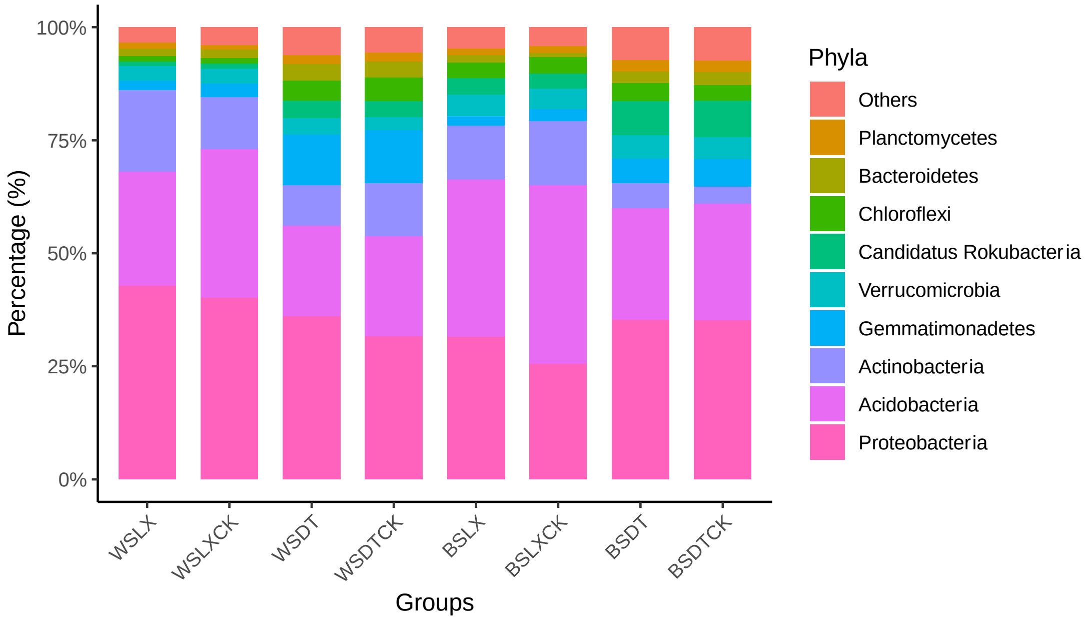

Supplementary Figure 2 the composition of prokaryotes in all samples

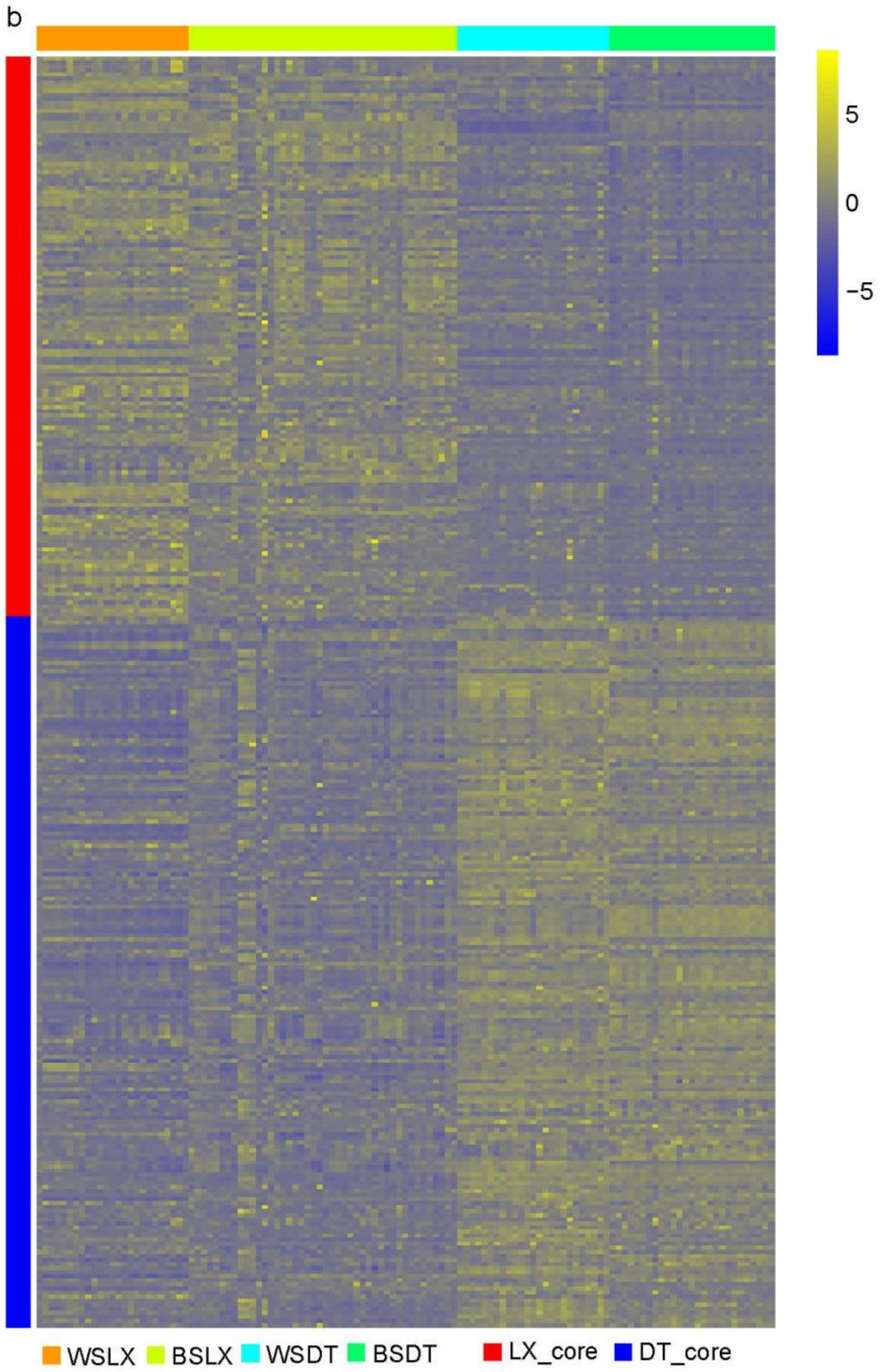

Supplementary Figure 3 The relative abundance of LX-core and DT-core KOs for rhizosphere microbiome involved in two-component system. Scale, relative abundance of KO at row normalization by removing the mean (centering) and dividing by the standard deviation (scaling). The color from blue to yellow represents a relative abundance of each KO from low to high.

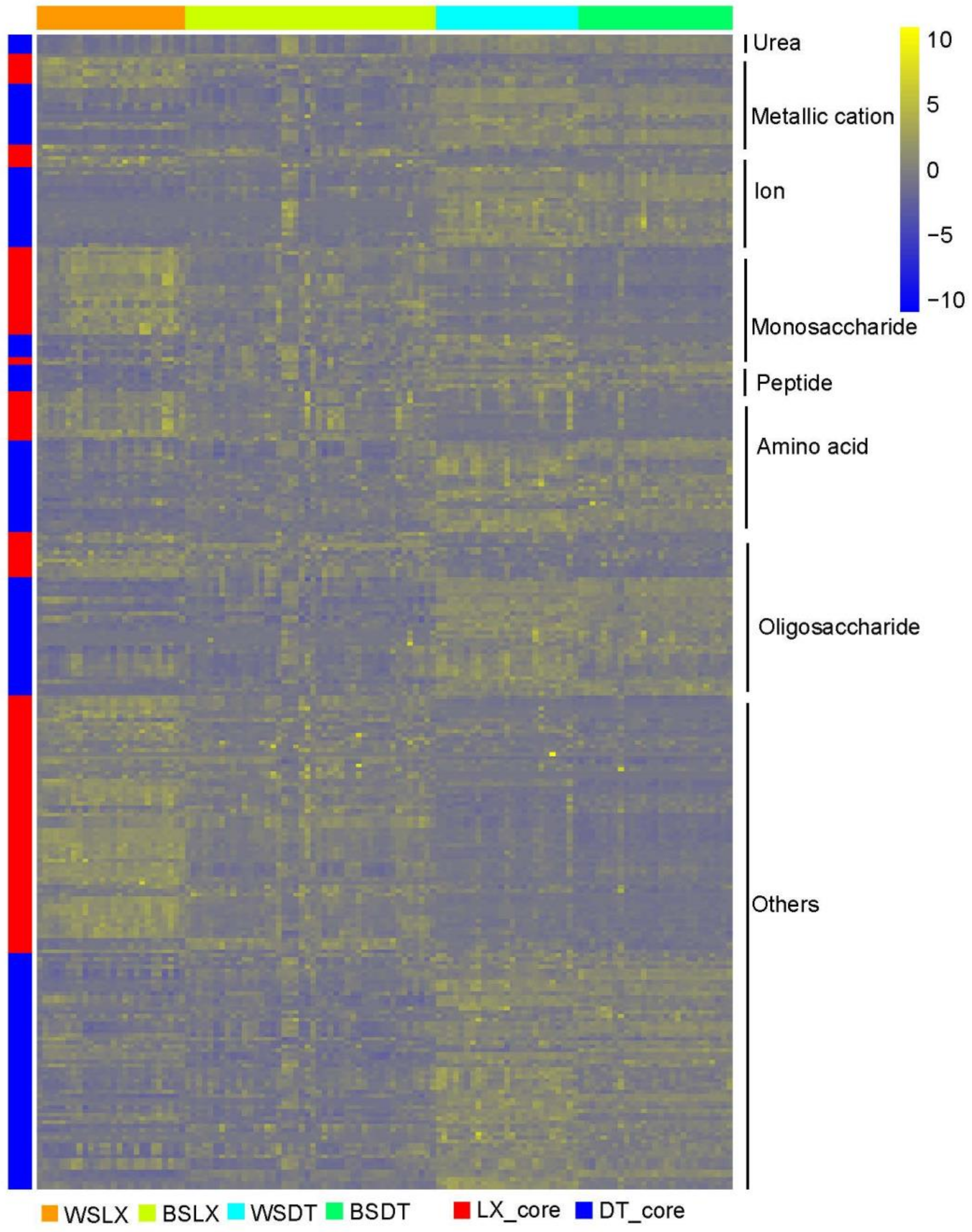

Supplementary Figure 4 The relative abundance of LX-core and DT-core KOs for rhizosphere microbiome involved in ABC transporters. Scale, relative abundance of KO at row normalization by removing the mean (centering) and dividing by the standard deviation (scaling). The color from blue to yellow represents a relative abundance of each KO from low to high.

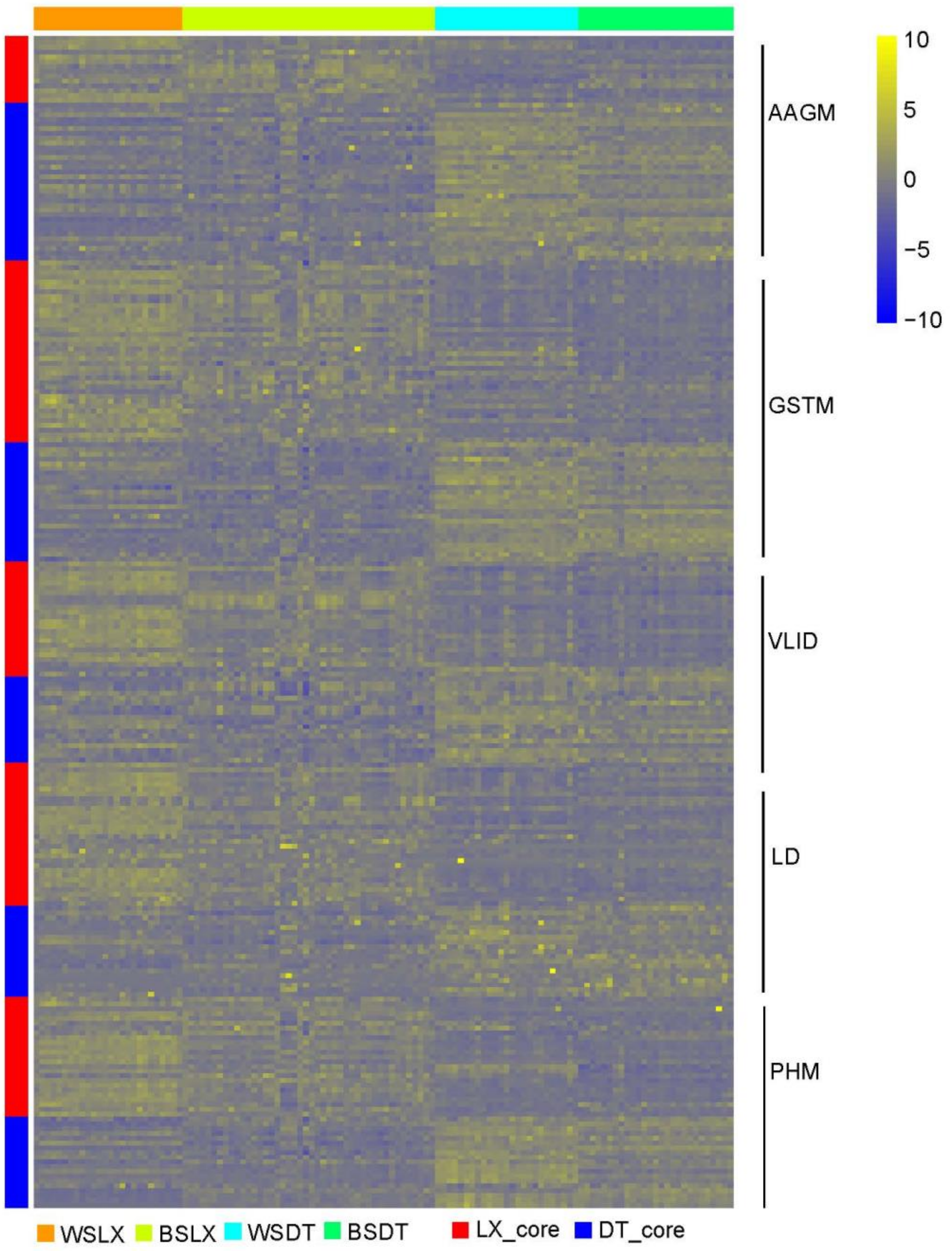

Supplementary Figure 5 The relative abundances of core rhizosphere KOs in different planting patterns involved in amino acid degradation. Scale, relative abundance of KO at row normalization by removing the mean (centering) and dividing by the standard deviation (scaling). The color from blue to yellow represents a relative abundance of each KO from low to high. AAGM Alanine, aspartate and glutamate metabolism, GSTM Glycine, serine and threonine metabolism, VLID Valine, LD leucine and isoleucine degradation, PHM phenylalanine metabolism.

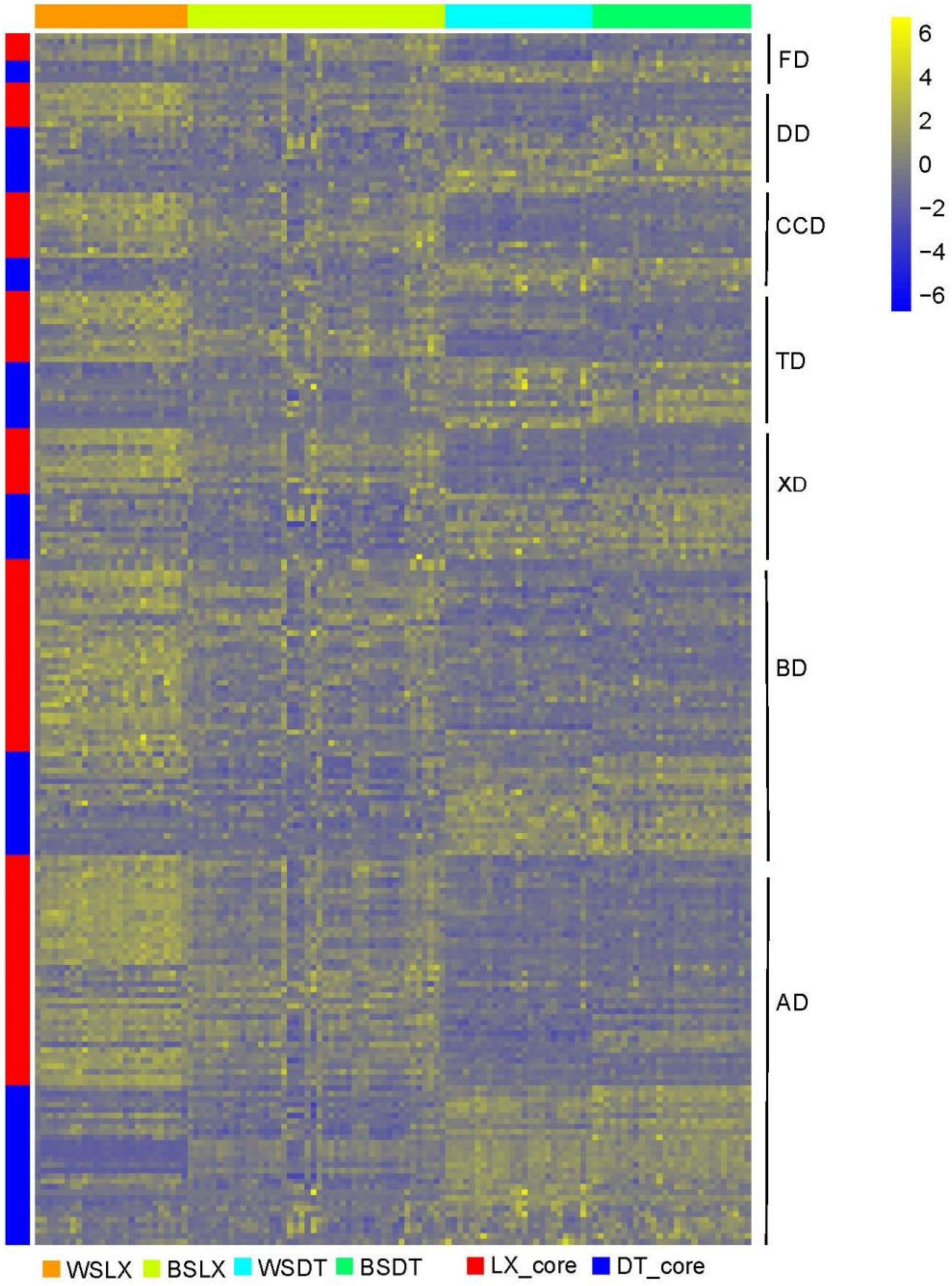

Supplementary Figure 6 The relative abundances of core rhizosphere KOs in different planting patterns involved in xenobiotics biodegradation and metabolism. Scale, relative abundance of KO at row normalization by removing the mean (centering) and dividing by the standard deviation (scaling). The color from blue to yellow represents a relative abundance of each KO from low to high. FD Fluorobenzoate degradation, DD Dioxin degradation, CCD Chlorocyclohexane and chlorobenzene degradation, TD Toluene degradation, XD Xylene degradation, BD Benzoate degradation, AD Aminobenzoate degradation.

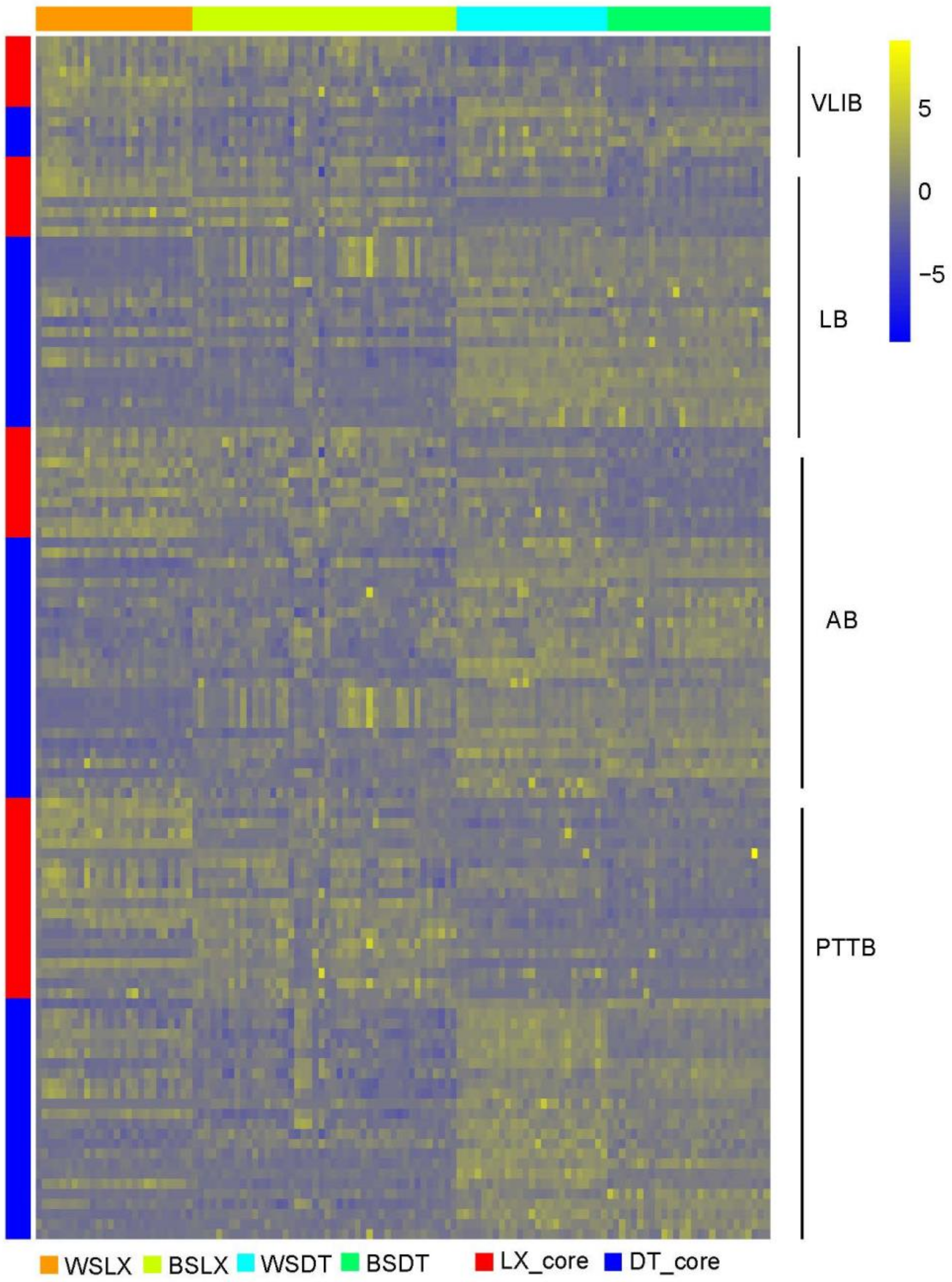

Supplementary Figure 7 The relative abundances of core rhizosphere KOs in different planting patterns involved in amino acid biosynthesis. Scale, relative abundance of KO at row normalization by removing the mean (centering) and dividing by the standard deviation (scaling). The color from blue to yellow represents a relative abundance of each KO from low to high. VLIB Valine, leucine and isoleucine biosynthesis, LB Lysine biosynthesis, AB Arginine biosynthesis, PTTB Phenylalanine, tyrosine and tryptophan biosynthesis.

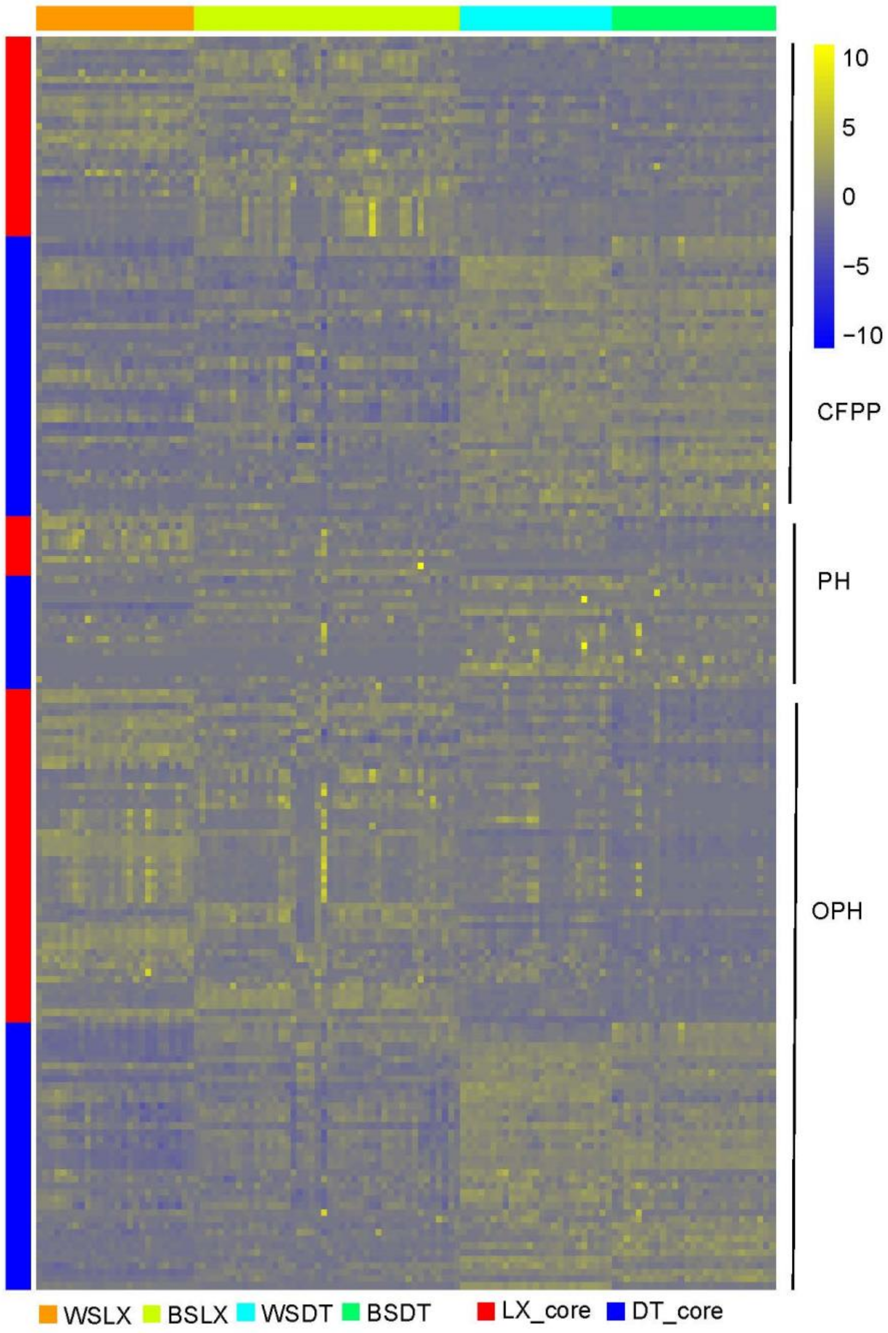

Supplementary Figure 8 The relative abundances of core rhizosphere KOs in different planting patterns involved in amino acid biosynthesis. Scale, relative abundance of KO at row normalization by removing the mean (centering) and dividing by the standard deviation (scaling). The color from blue to yellow represents a relative abundance of each KO from low to high. VLIB Valine, leucine and isoleucine biosynthesis, LB Lysine biosynthesis, AB Arginine biosynthesis, PTTB Phenylalanine, tyrosine and tryptophan biosynthesis. CFPP Carbon fixation pathways in prokaryotes, PH Photosynthesis, OPH Oxidative phosphorylation.

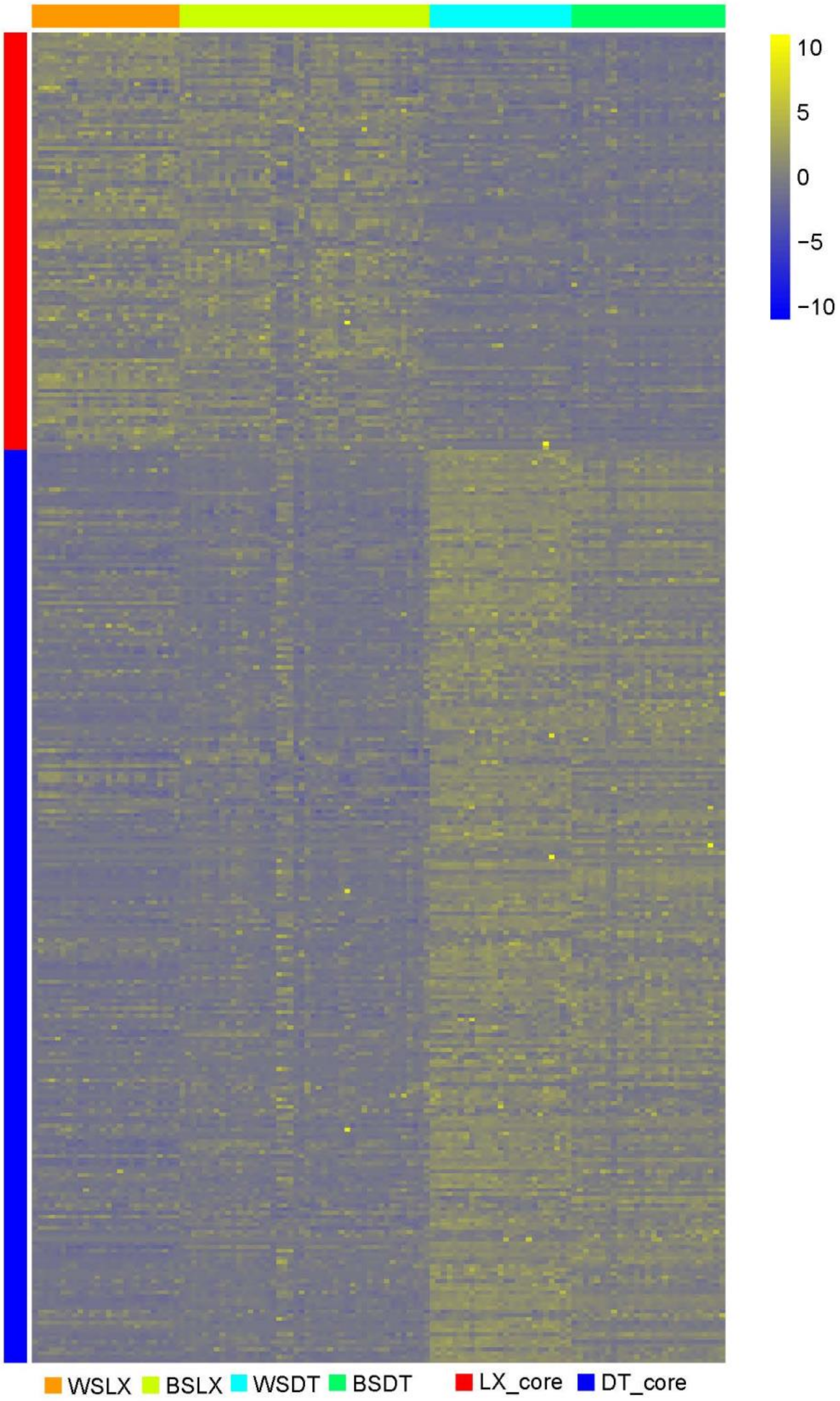

Supplementary Figure 9 The relative abundances of core rhizosphere KOs in different planting patterns involved in peptidases. Scale, relative abundance of KO at row normalization by removing

the mean (centering) and dividing by the standard deviation (scaling). The color from blue to yellow represents a relative abundance of each KO from low to high.

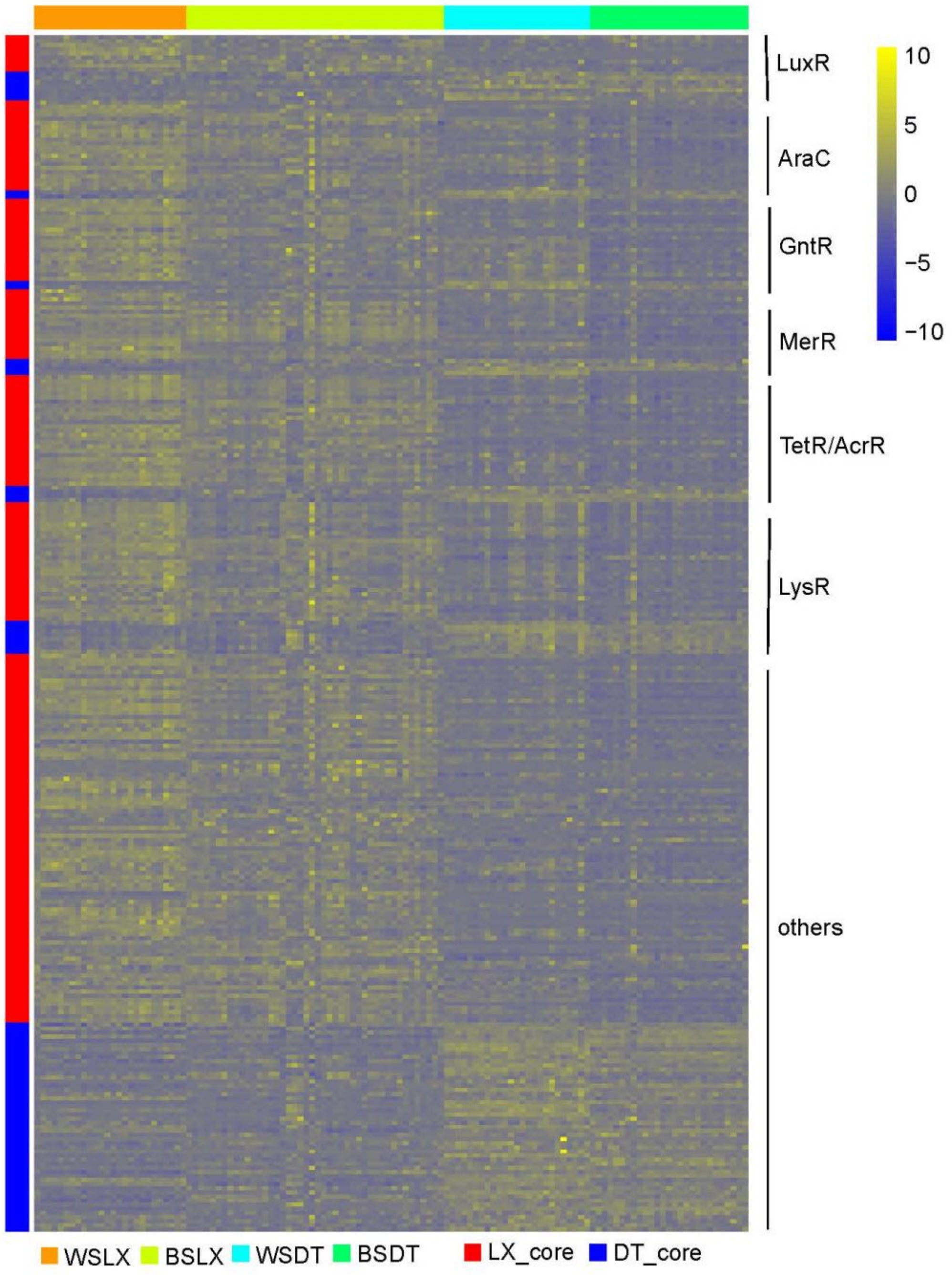

Supplementary Figure 10 The relative abundances of core rhizosphere KOs in different planting patterns involved in transcription factors. Scale, relative abundance of KO at row normalization by removing the mean (centering) and dividing by the standard deviation (scaling). The color from blue to yellow represents a relative abundance of each KO from low to high.
